# Research Update: Materials design of implantable nanogenerators for biomechanical energy harvesting

**DOI:** 10.1063/1.4978936

**Published:** 2017-03

**Authors:** Jun Li, Xudong Wang

**Affiliations:** Department of Materials Science and Engineering, University of Wisconsin-Madison, Madison, Wisconsin 53706, USA

## Abstract

Implantable nanogenerators are rapidly advanced recently as a promising concept for harvesting biomechanical energy *in vivo*. This review article presents an overview of the most current progress of implantable piezoelectric nanogenerator (PENG) and triboelectric nanogenerator (TENG) with a focus on materials selection, engineering, and assembly. The evolution of the PENG materials is discussed from ZnO nanostructures, to high-performance ferroelectric perovskites, to flexible piezoelectric polymer mesostructures. Discussion of TENGs is focused on the materials and surface features of friction layers, encapsulation materials, and device integrations. Challenges faced by this promising technology and possible future research directions are also discussed.

## I. INTRODUCTION

Since the first clinical implantation of pacemaker in 1958, implantable medical devices (IMDs) have experienced a rapid evolution.^1–5^ Take the implantable cardioverter defibrillator as an example, the number of implantations increased more than 10 times from 1990 to 2002.^6^ Generally, the trends of IMDs evolution are leading toward smaller, lighter, and longer life span. The electronic components of IMDs can now be made in micrometer size owing to the rapid development of modern semiconductor industry. Nevertheless, their battery systems are far lagging behind, which contribute the majority of weight and volume (up to 90%) of the implants. Additional surgeries may be needed to replace the drained batteries for IMDs. Additionally, the battery chemistry is often the most dangerous and toxic component in an implantable system. Development of proper implantable battery technology is at a critical moment for the next generation IMDs that possess cell-level sizes, tissue-like soft structures, extreme reliability, and powerful and versatile functionalities.

Harvesting energy *in vivo* has recently attracted considerable attention because of its promising self-sufficient powering ability.^7–9^ The concept of human-powered computing, presented by IBM in the 1990s, illustrated the availability of energy in the human body.^10^ Breath, exhalation, blood flow, and heart beating can produce power in the range of ~0.3–1 W. Given *μ*W-level power consumption for typical sensing nodes and mW-level for wireless communication, energy available within a human body is sufficient to support the operation of implantable devices.^11^ In 2006, Nanogenerator (NG) emerged as a promising strategy to harvest mechanical energy from ambient environment.^12,13^ NGs utilize nanostructured functional materials to generate electricity from physical displacement via the effect of either piezoelectric (PENG) or triboelectric (TENG). The NG technology opens a new route toward efficient micro-/nano-scale mechanical energy harvesting that is suitable for implantable systems. Compared to other technologies, such as biofuel cells or thermoelectrics, both PENG and TENG offer an excellent energy conversion efficiency, a much simpler configuration and can be made with high flexibility.^14–19^ Driven by the promising application potentials in biomedical engineering, flexible PENGs and TENGs have experienced rapid developments in recent years for *in vivo* biomechanical energy harvesting. The *in vivo* application imposes stringent requirements to the NG designs particularly on device reliability, operation safety, and materials biocompatibility. Although there have been numbers of comprehensive review articles on the progress of PENGs and TENGs,^20–25^ no focused review has been dedicated to the i-NGs. To address this emerging literature need, this review article provides an overview of the most recent progress in i-NGs with a focus on materials selection, engineering, and assembly. Both PENG and TENG are covered. Potential *in vivo* applications, such as self-powered pacemaker and healthcare monitor, are also discussed. Moreover, critical challenges that are faced by this promising technology are analyzed and possible research directions are suggested.

## II. MATERIALS DESIGN FOR IMPLANTABLE PIEZOELECTRIC NANOGENERATORS (i-PENG)

Piezoelectric materials are a class of materials that can generate electric polarization in response to mechanical stress. The first NG was made from piezoelectric ZnO nanowire (NW) arrays, where deflecting the NWs could convert nanoscale mechanical energy into electricity.^12^ This new nanoscale powering technology processes great promises for implantable biomedical device owing to the small size, good flexibility, and appreciable energy output. After the first attempt to implant ZnO-based PENGs inside rats, i-PENGs consisting of various piezoelectric materials (e.g., ZnO, PZT, BZT-BCT, and PVDF) have been designed and implemented for biomedical applications.^25–33^

### A. ZnO PENGs

ZnO has good biocompatibility and biosafety, which is essential for their applications in biomedical engineering. The biodegradability and biocompatibility of ZnO wires have been demonstrated by Zhou *et al.* through investigating the interaction of ZnO wires with horse blood serum.^34^ The ZnO wires immersed in horse blood serum showed obvious degradation after an hour and it would eventually dissolve and be absorbed by the body as nutrition. Later Wang *et al.* further investigated the cellular level biocompatibility and biosafety of ZnO NWs.^35^ Two cell lines, Hela cell line and L-929 cell line, were selected for the biocompatibility study by planting the cells in a 96-well plate filled with ZnO NW-phosphate buffer suspensions for different incubation times (12–48 h). The test showed that when concentration of ZnO NWs was less than 10 *μ*g/L, there was no obvious reduction in the viability of both Hela and L-929 cells, confirming the good biocompatibility and biosafety of ZnO NWs.

**FIG. 1:**
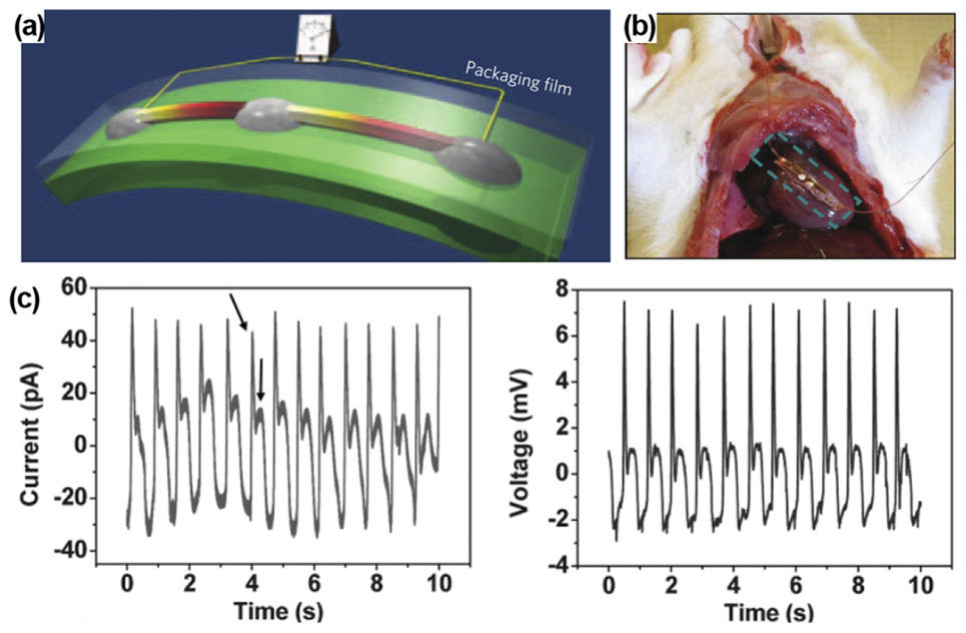
(a) Schematic of ZnO NW-based NG on a flexible substrate. Reprinted with permission from Yang *et al.*, Nat. Nanotechnol. **4**(1), 34 (2009). Copyright 2009 Springer Nature. (b) ZnO NG attached on the heart of a rat. (c) Output current and voltage profiles obtained from the ZnO NG under regular heart beating. Reprinted with permission from Li *et al.*, Adv. Mater. **22**(23), 2534 (2010). Copyright 2010 Wiley-VC.

The first *in vivo* implementation of ZnO-based NG for biomechanical energy harvesting was demonstrated by Li *et al.* in 2010.^26^ In this design, a ZnO NW was fixed on a flexible polyimide substrate and covered by a flexible polymer. The polymer package isolated the ZnO NW from the bio-fluid environment (Fig. 1(a)). Adult rats were used as experimental subjects and the NG was attached to the surface of heart (Fig. 1(b)). When the NG was applied on heart, the average open-circuit voltage (V_oc_) and short-circuit current (I_sc_) were 3 mV and 30 pA, respectively, under normal heart beating (Fig. 1(c)). Driven by breathing, the V_oc_ and I_sc_ were 1–2 mV and 1–4 pA, respectively. This was the first successful demonstration of NGs under *in vivo* conditions to convert biomechanical energy into electricity.

Nevertheless, there were not many follow-up reports on ZnO i-PENG after the first successful demonstration. The main reason might be the extremely low electric outputs that were not compatible to any biomedical devices. In addition, lacking of flexibility, stability, and scalability in ZnO nanostructure fabrication and manipulation for i-PENG development also limited its applications in practical biomedical systems. While the implantable NG concept remained intriguing, research shifted to exploring new materials or designs that have higher piezoelectric response while still holding the nanoscale geometric merits.

### B. Perovskite-structured PENG

Compared to ZnO, perovskite-based ferroelectric materials exhibit much higher piezoelectric coefficients and they typically produce much higher electric outputs in bulk.^9,36,37^ As the most broadly used ferroelectric material, lead zirconate titanate (PZT) was first fabricated into the structure of nanofibers for NG development.^38^ A voltage of 1.6 V and power of 0.03 *μ*W were obtained, which were 2–3 orders of magnitude higher than the original ZnO-based NGs.

**FIG. 2:**
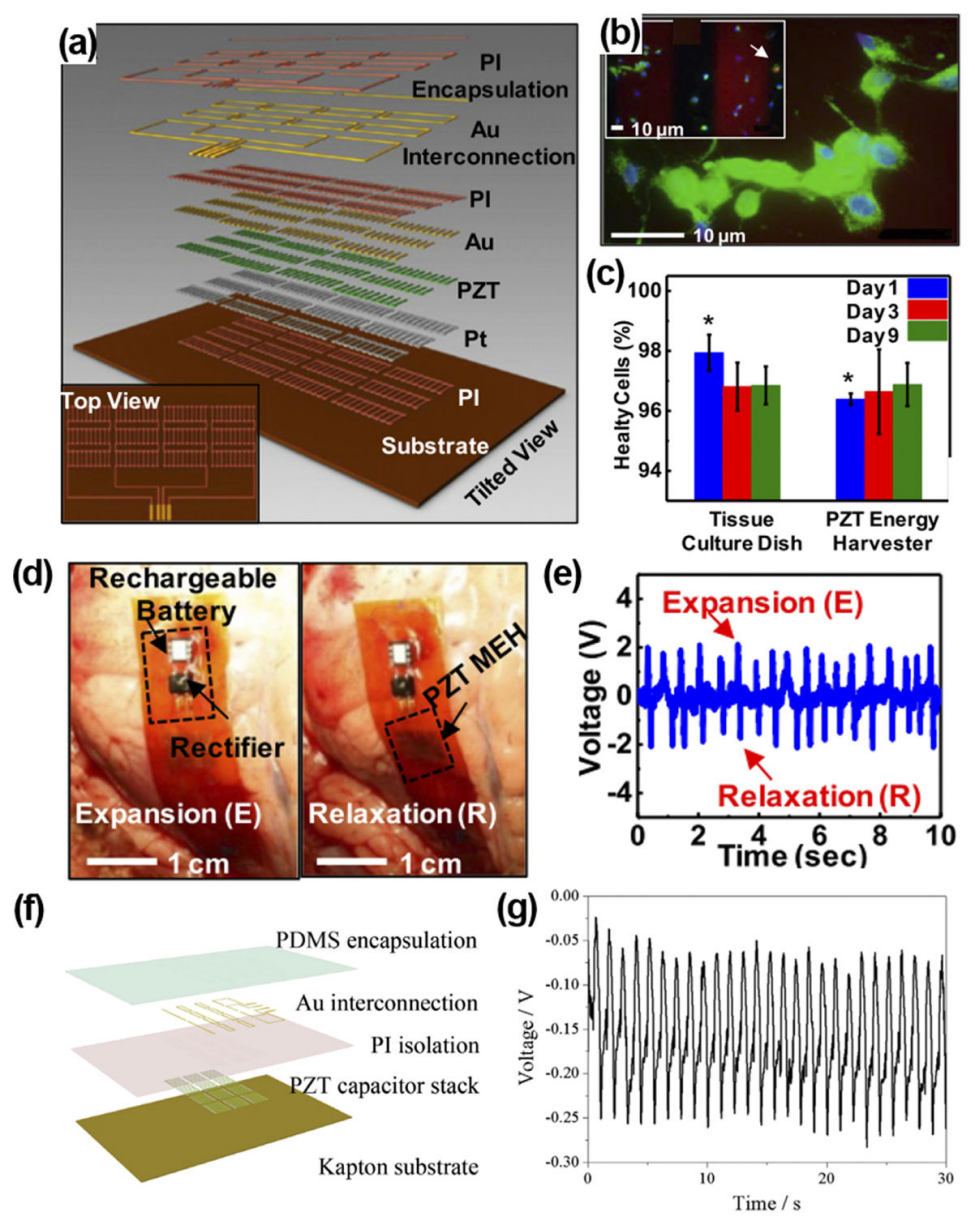
(a) Schematic illustration of a PZT nanofiber-based flexible NG. (b) Fluorescent image of a live/dead assay of rat smooth muscle cells on NG surface. Green, blue, and red colors represent live cells, intact nucleus, and dead cells, respectively. Inset shows dead cells pointed by the arrow. (c) Comparison between cells grown on device and cells grown on culture plates. ((d) and (e)) PZT NG attached to the heart of bovine (d) and corresponding output voltage (e). Reprinted with permission from Dagdeviren *et al.*, Proc. Natl. Acad. Sci. U. S. A. **111**(5), 1927 (2014). Copyright 2014 National Academy of Sciences. (f) Schematic structure of PZT thin film-based implantable NG. (g) Voltage outputs of the PZT i-PENG driven by heart beating when attached to the swine heart. Reprinted with permission from Lu *et al.*, Sci. Rep. **5**, 16065 (2015). Copyright 2015 Springer Nature.

In 2014, Dagdeviren *et al.* demonstrated the first flexible and implantable NG based on PZT nanoribbons.^28^ The PZT ribbons were covered with Cr/Au and Ti/Pt electrodes, connected by Au interconnection, and encapsulated by 1.2 *μ*m polyimide (Fig. 2(a)). The biocompatibility of the PZT-based NG was evaluated by planting the rat’s smooth muscle cells on top of packaged NG surface. The cells well adhered to the device and 96% cells were healthy after 9 days (Fig. 2(b)). Moreover, there was no difference of viability between cells grown on the PZT NG and those grown on tissue culture plates (Fig. 2(c)). The *in vivo* performance of the PZT NG was investigated by affixing it to the bovine and ovine hearts (Fig. 2(d)). The NG generated an output voltage up to 4 V driven by heart beating when the animal was put into anesthesia (Fig. 2(e)). A similar high voltage of ~4 V was also produced when the NG was mounted on the lung of bovine. Later, Lu *et al.* developed a similar PZT ribbon NG and performed *in vivo* test when the swine was awake from anesthesia, which was closer to the practical scenario.^29^ The PZT ribbon was printed on a flexible Kapton substrate and the entire device was packaged by PDMS (Fig. 2(f)). The NG was sutured between the left ventricular and right ventricular of heart. The peak-to-peak voltage reached 3 V at the open chest under normal heart beating. They found that the voltage output dropped to only 0.3 V after the chest was closed and the swine was in conscious condition, while the output was still regular and stable (Fig 2(g)). The significant reduction in voltage outputs was probably because when the chest was closed, the intrathoracic pressure was increased and surrounding tissues became closer, and thus the deformation amplitude of the NG was largely limited.

**FIG. 3:**
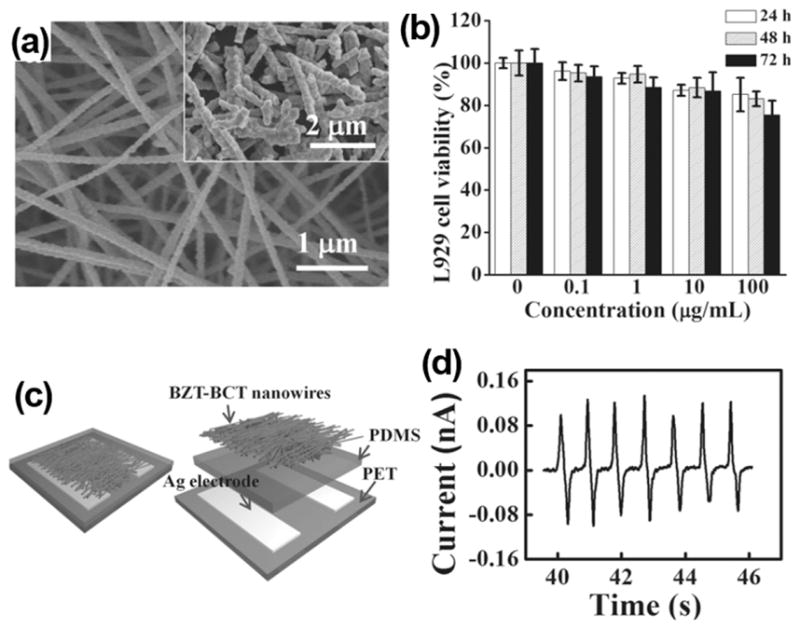
(a) SEM image of as-prepared BZT-BCT NWs. The inset is NWs after being grinded. (b) L929 cell viability as a function of time and concentration of BZT-BCT NWs. (c) Schematic image and (d) current outputs of BZT-BCT NWs based NG. Reprinted with permission from Yuan *et al.*, Adv. Mater. **26**(44), 7432 (2014). Copyright 2014 Wiley-VCH.

Although PZT-based NGs can generate much higher outputs than most previously reported NGs, one critical problem is the involvement of lead element, which is toxic and would cause damage to both human body and environment. Lead-free materials with piezoelectric coefficients comparable to PZT were then studied as promising alternatives for the development of i-NGs. 0.5Ba(Zr_0.2_Ti_0.8_) O_3_-0.5(Ba_0.7_Ca_0.3_)TiO_3_ (BZT-BCT) is a high-performance lead-free ferroelectric material with a piezoelectric coefficient of 620 pC/N. BZT-BCT NWs were fabricated by an electrospinning method^39^ and used for i-NG development.^27^ The as-synthesized BZT-BCT NWs had an average diameter of 210 nm and the NWs were grinded into nanorods with 0.2 to 5 *μ*m in length (Fig. 3(a)). For biocompatibility test, Chang liver cells and L929 cell were cultured in a culture solution containing BZT-BCT NWs. The viability of cells demonstrated that within large range of concentration, BZT-BCT NWs had no harmful influence on both types of cells (Fig. 3(b)). A BCT-BZT NG was fabricated by applying a thin layer of BCT-BZT NWs in between two Ag electrodes on a PET substrate and packaged by PDMS (Fig. 3(c)). The NG was attached to the subcutaneous tissue on the back of a rabbit to test the biocompatibility. After 5 weeks of implantation, the rabbits lived well and there was no tissue damage around the implantation. An output current of 0.13 nA could be generated by the implanted NG without noticeable signal decay over the implantation period (Fig. 3(d)). BZT-BCT NWs were also composited with PDMS into a bulk film that produced *in vivo* V_oc_ of 1.2 V and I_sc_ of 0.51 *μ*A inside the rabbit.^30^

In general, perovskite ferroelectric materials could produce high electric output *in vivo* which essentially made the NG concept practical for powering biomedical devices. However, toxicity (inclusion of lead elements) is the major concern of using this type of materials for *in vivo* application. Development of lead-free perovskite ferroelectric materials is a promising solution to this problem and it has been an active research field in general. With the continuous emergence of new high-performance lead-free piezoelectric materials, better material solutions to i-NG design may come as well. It also should be noted that the complex element combinations may also raise additional challenges in mass production and biocompatibility evaluation.

### C. PVDF PENGs

Other than ceramic-based piezoelectrics, polymer-based piezoelectric materials are intuitively more suitable to biological systems. β-phase polyvinylidene fluoride (PVDF) is the most broadly used piezoelectric polymer that has been applied in many flexible electromechanical systems.^40–43^ The piezoelectric coefficients of β-phase PVDF were found in the range of ~20–30 pC/N (d_33_) and ~16–18 pC/N (d_31_), which are sufficient to generate useful electric outputs.^44^ The first PVDF NG was demonstrated using electro-spun nanofibers shortly after the invention of ZnO-based NGs with a small electric output (~10–20 mV).^45^

The biocompatibility of PVDF has been initially investigated by Douville *et al.* Sterilized PVDF sutures were used to anastomose a pre-clotted polyester vascular prosthesis as a thoracoabdominal bypass in eight adult mongrel dogs. The long-term (up to 6 months) tissue responses were examined by histologic and scanning electron microscopy after implantation. During the implantation period, only a few lymphocytes and macrophages (inflammatory cells) and little neocollagen were observed around the PVDF sutures, which confirmed the excellent biocompatibility of PVDF.^46^

**FIG. 4:**
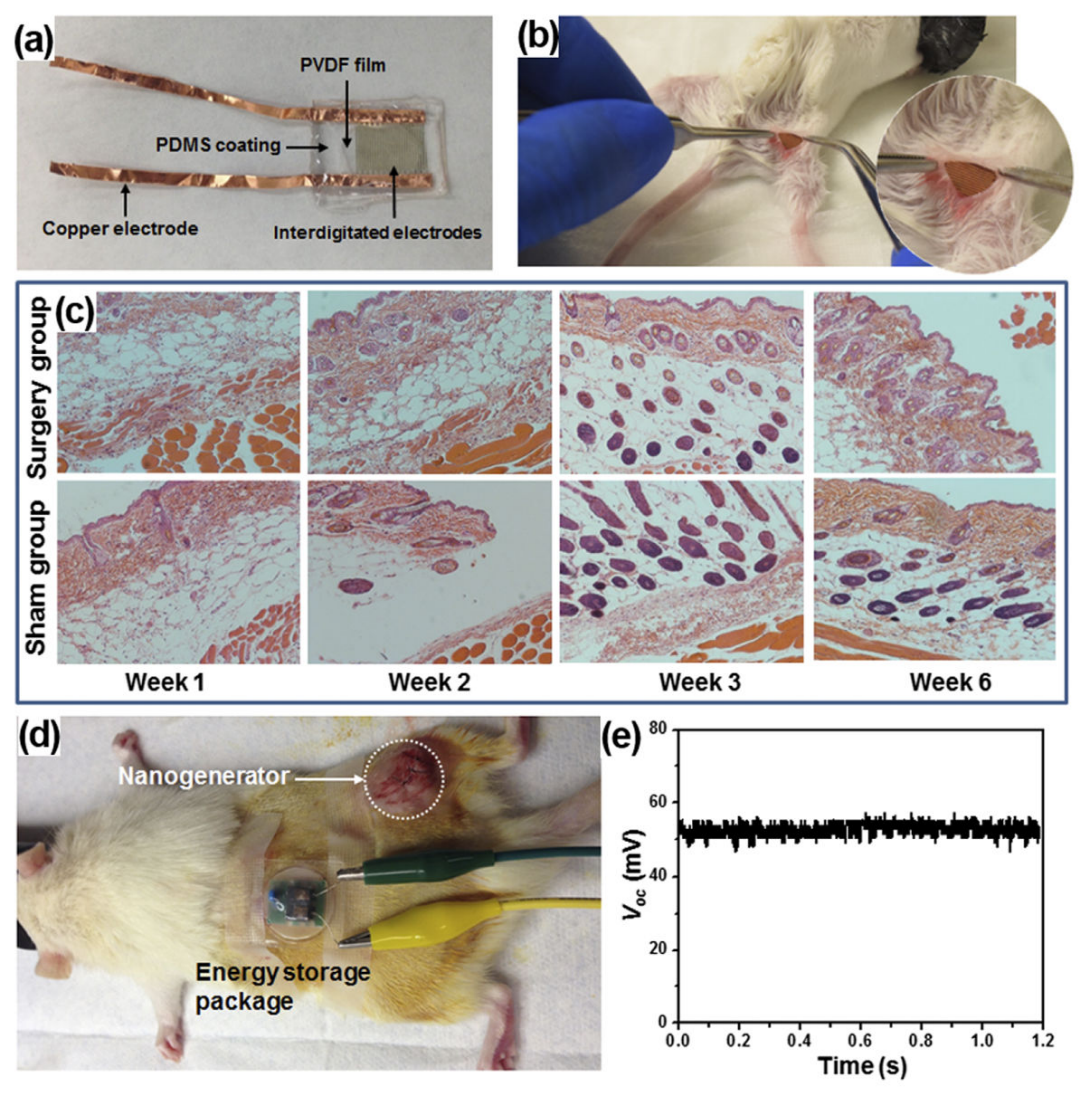
(a) A packaged PVDF NG. (b) A surgery picture of the mouse with the PVDF NG embedded. (c) Surgical pathology analysis of the NG-implanted and sham group. (d) Setup of the *in vivo* electricity generation and storage in a rat. (e) DC voltage output from the NG-charged energy storage package.

Recently, Yu *et al.* systematically studied the biocompatibility and *in vivo* operation of a mesoporous PVDF-based flexible NGs. The NG was built with a sponge-like mesoporous PVDF film. The PVDF film was coated with a pair of interdigitated gold electrodes for piezoelectric charge collection, and the entire structure was packaged inside PDMS (Fig. 4(a)). To evaluate the biocompatibility, a packaged PVDF NG was inserted under the skin of mice’s right leg (Fig. 4(b)). The mice with implanted NG were sacrificed at week 1, 2, 3, and 6 for histological examination. Surgery and sham control groups were extracted from the right and left legs of the animal, respectively. Surgical pathology analysis revealed no overall differences between the NG-implanted and sham thigh in terms of skin histology (Fig. 4(c)). There were limited inflammatory infiltrates in both groups. In addition, there were no signs of cellular toxicity or muscular atrophy/degeneration in tissue sections. Overall, there were no signs of toxicity or incompatibility induced by the NG in mice, suggesting excellent biocompatibility of the PDMS-packaged PVDF NG system.

The *in vivo* biomechanical energy harvesting experiments were conducted in living rats by implanting a capsulated NG between the epithelial and muscle layer. The NG was left inside the rats for 5 days to examine the time-dependent durability and performance in an *in vivo* environment. Voltage was measured repeatedly every day while the rats were housed under normal conditions. The peak voltages were determined to be 0.26 ± 0.03 V, 0.20 ± 0.03 V, 0.23 ± 0.02 V, 0.21 ± 0.04 V, and 0.23 ± 0.05 V for day 1–5, respectively. The stable voltage output suggested that the NG could function normally without noticeable decay, despite the constant motion of the rat during the test.

An energy storage system comprised of a miniaturized bridge circuit and a ceramic capacitor to demonstrate the capability of the PVDF NG in charging or powering small electronics *in vivo*. The output leads of the implanted NG were wired through the epithelial and muscle layer in the thigh and back region and extended out from the back of the rat where the motion is minimized. The energy storage pack was connected through the leads and fixed on the back of the rat (Fig. 4(d)). Upon the movement of the rat’s leg, the NG was straining and generating electricity. After rectification, the alternating current (AC) was converted to direct current (DC) signal without observable loss through the *in vivo* electricity transport. The peak value of voltage after rectification reached about 200 mV. This output was converted to a 52 mV DC voltage through a 1 *μ*F capacitor (Fig. 4(e)), suggesting a good potential of using NG to power biomedical electronics *in vivo*.

**FIG. 5:**
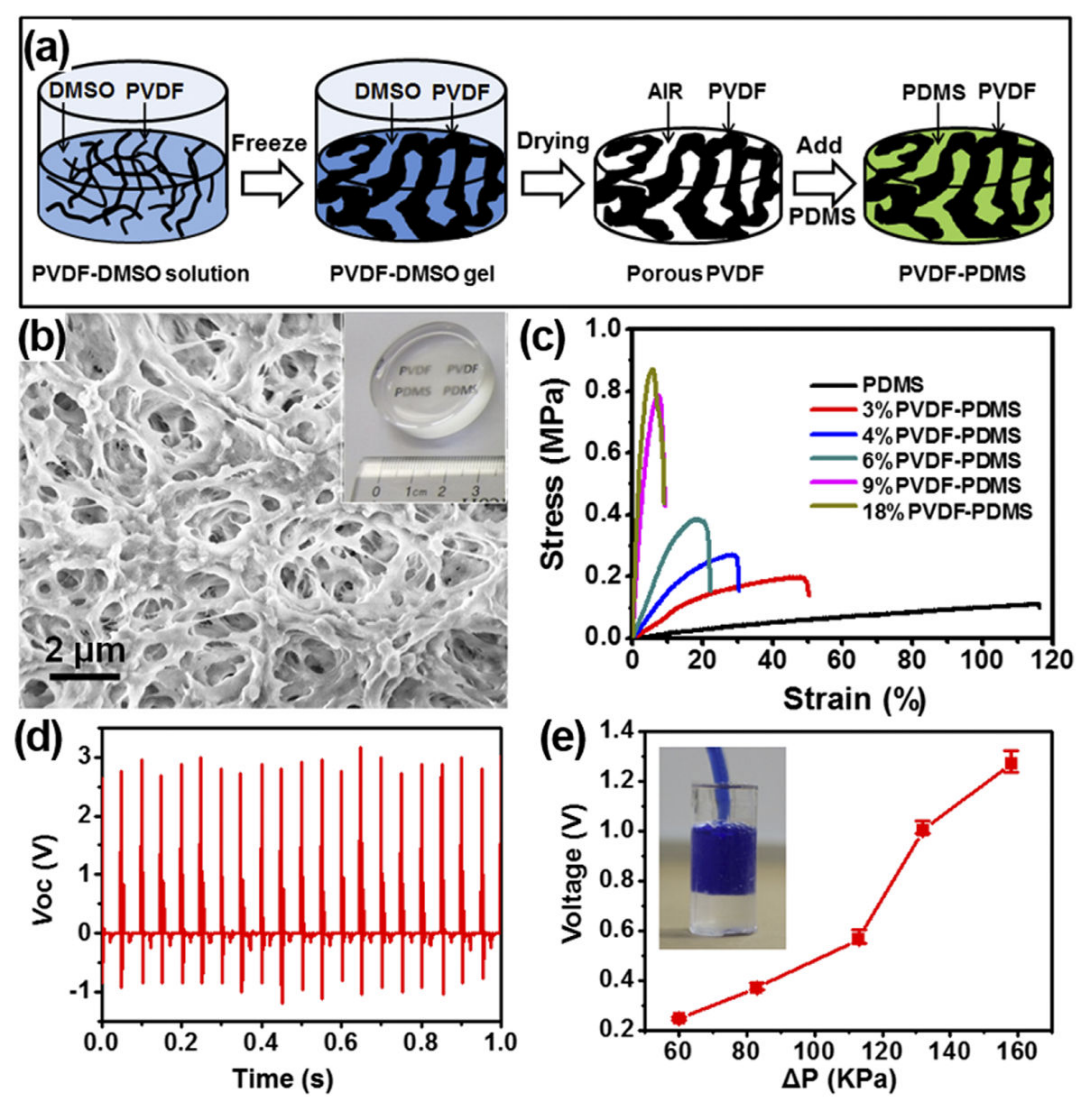
(a) Schematic procedure of preparing mesoporous PVDF-PDMS composite films. (b) SEM image showing the high porosity of the PVDF film. Inset is a phone of a PVDF-PDMS composite film. (c) Tensile stress/strain curves of PVDF-PDMS films with different PVDF volume fractions. (d) Piezoelectric voltage profiles measured from a 4% PVDF-PDMS NG slab. (e) Voltage obtained from the imbedded NG as a function of internal pressure difference.

While the polymer piezoelectric is naturally much softer than ceramic, its mechanical modulus is still 1–2 orders of magnitude higher than normal human muscles and tissues. It is necessary to further reduce its modulus to the same level of organic tissues in order for practical *in vivo* applications with minimal side impacts to normal body functions. Mesoporous PVDF films were thus developed with tunable mechanical modulus for effective *in vivo* biochemical energy harvesting.^47^ The mesoporous PVDF was fabricated by a sol-gel process as schematically illustrated in Fig. 5(a). The as-received PVDF surface (4% PVDF) exhibited an interconnected network feature with observable pore size from nearly one micron to hundred nanometers (Fig. 5(b)). Through this approach, mesoporous PVDF networks were fabricated with a wide range of PVDF volume ratio from 3% to 18%. The as-prepared mesoporous PVDF slabs were white, foam-like, and can be made in large scale. The white porous PVDF slabs turned into transparent after being infiltrated with PDMS (inset of Fig. 5(b)) and the transparency slightly decreased when the PVDF volume fraction was above 9%.

Infiltration of PDMS could largely improve PVDF films’ elasticity and tune their mechanical strength. The tensile and compressive tests were conducted on the PVDF/PDMS composite films at a displacement rate of 20 mm/min. All the samples exhibited elastomer-type behavior, while increasing the PVDF fraction significantly raised the material’s stiffness. The fracture strain rapidly decreased from ~50% at 3% PVDF to <10% when the PVDF fraction was greater than 9% (Fig. 5(c)). The tensile moduli were calculated from the linear elastic region of stress/strain curves. The tensile modulus increased from 0.8 to 30.16 MPa with the increase of PVDF volume fraction. This modulus range covered the modulus of certain human organs, such as blood vessels.

The tunable mechanical property endowed unique advantages to the piezoelectric polymer composite slabs for mechanical energy harvesting. NGs were fabricated from the porous PVDF/PDMS films and their electric outputs were measured under a force of 6 N and at a frequency of 20 Hz, which gave a pressure of 0.3 MPa. Under this condition, the average peak values of the voltage and current were found to be about 2.87 V and 3.42 *μ*A, respectively (Fig. 5(d)). Even with a significantly less PVDF fraction due to the highly porous structure, the obtained output voltage and current were still comparable or even higher than that of other PVDF-based NGs reported previously. An artificial blood vessel model was made using PDMS with a PVDF-PDMS NG imbedded inside the wall to demonstrate the potential of harvesting energy from blood pressure change (inset of Fig. 5(e)). Under a series of ΔP applied to the artificial arterial system at a constant rate of 60/min, the average peak-to-peak values of the voltage raised from 0.25 V to 1.27 V when ΔP increased from 60 KPa to 158 KPa (Fig. 5(e)).

**FIG. 6:**
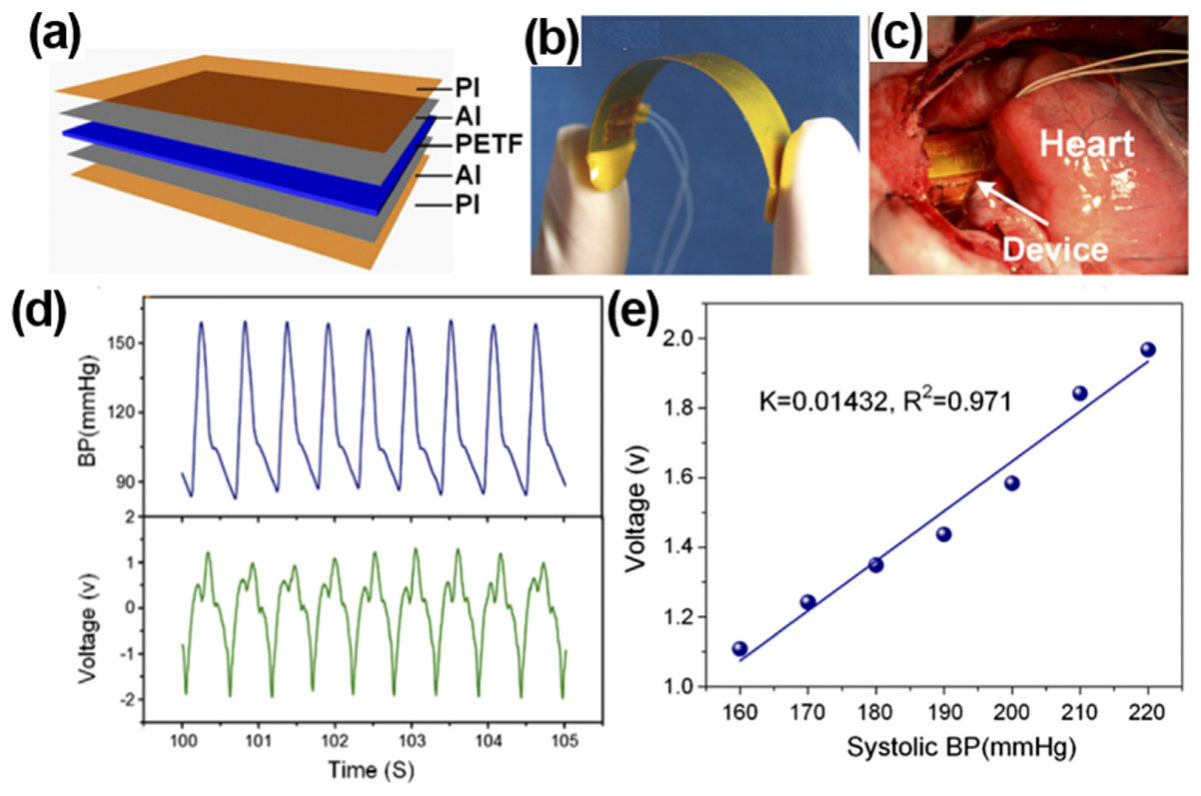
(a) Schematic image of PI-packaged PVDF piezoelectric NG. (b) Photograph of the flexible NG device. (c) A flexible NG wraps around the ascending aorta of a porcine. (d) Blood pressure of the porcine (top) and corresponding output voltage of the NG (bottom) as a function of time. (e) A plot of NG output voltage as a function of blood pressure showing a good linearity. Reprinted with permission from Cheng *et al.*, Nano Energy **22**, 453 (2016). Copyright 2016 Elsevier.

Owning to its outstanding flexibility, PVDF thin films could be wrapped around the aorta and harvest energy from pulsation. Zhang *et al.* prepared implantable PVDF thin film NGs and studied the *in vitro* and *in vivo* performance.^25,31^ The PVDF NG was designed by sandwiching a polarized 200 *μ*m PVDF thin film between aluminum electrodes (Fig. 6(a)). Biosafe silicone-packaged Cu leads were attached to the aluminum layer and 50 *μ*m polyimide films were utilized to encapsulate the entire device. The PVDF NG had a high flexibility (Fig. 6(b)) and was wrapped around the ascending aorta of porcine for *in vivo* test (Fig. 6(c)). Under 70 bpm heart rate and 160/105 mmHg blood pressure, the maximum V_oc_ and I_sc_ were 1.5 V and 300 nA, respectively (Fig. 6(d)). The electric outputs were linearly proportional to blood pressure with a slope of 14.3 mV/mmHg obtained from the Yorkshire porcine system (Fig. 6(e)). The excellent linearity between generated voltage signal and systolic blood pressure revealed a good potential of the piezoelectric films for real-time blood pressure monitoring.

In general, PVDF-based polymer NGs are a favorable design for biomedical application owing to their biocompatibility, flexibility, and good processability to create mesoscale structures with tunable mechanical moduli. They are promising for applications on IMDs. Nevertheless, no PVDF-based PENG has yet been integrated with biomedical devices. It is desirable to achieve higher output power in order to satisfy the power consumption requirements of practical systems.

## III. MATERIALS DESIGN FOR IMPLANTABLE TRIBOELECTRIC NANOGENERATORS (i-TENGs)

Several years after the invention of PENG, the triboelectric effect was introduced as a promising new concept for environmental mechanical energy harvesting.^48^ The triboelectric nanogenerator (TENG) operates based on the coupling of triboelectrification and electrostatic induction. A typical TENG consists of two dissimilar materials that have different electronegativities. Contact of these two materials causes them to gain or lose electrons, respectively, and induces triboelectric potential. Once separated, the change in potential would drive electron to flow through an external circuit as electricity output. So far, the record efficiency of TENG has exceeded 80%, which largely outperformed PENG devices and showed great potential in various applications.^49^ Owing to its high output and general usage of flexible polymer functional materials, TENG is very promising to be applied to biomedical applications. Although the TENG technology has just been demonstrated for a few years, numbers of implantable TENG (i-TENG) designs with good *in vivo* performance have been brought into reality. Here, we briefly overview the state-of-the-art i-TENG designs and technologies.

### A. Materials selections and designs for implantable TENG (i-TENG)

Since TENGs operate based on triboelectrification, it possesses much broader material selections compared to PENGs. Many flexible dielectric polymers can find a good application potential in TENG designs. For example, PTFE (Polytetrafluoroethylene) and PDMS were usually used as the triboelectric layers in the implantable TENGs,^15,50–52^ because of their strong electron affinity among the materials listed in the triboelectric series and excellent flexibility and biocompatibility. As for charge collection, aluminum is usually employed as both triboelectric layer and electrode in i-TENGs.^50–52^ It has strong tendency to lose electrons when contacted with positive polymer materials. Aluminum also has very high conductivity and relatively small elastic modulus, which allows the i-TENG to remain at good flexibility after integration.

**FIG. 7:**
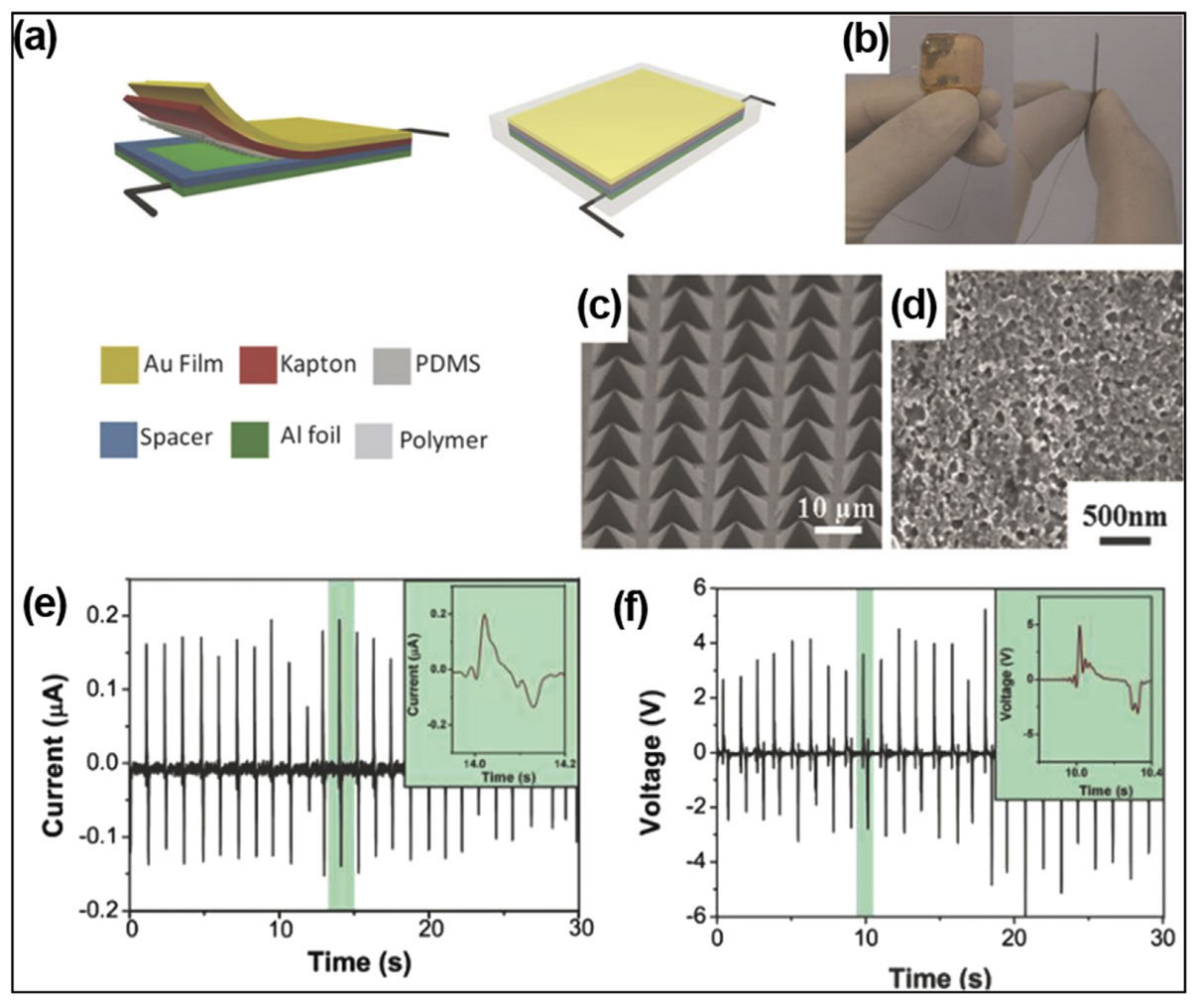
(a) Schematic of an i-TENG made from PDMS and Al friction films (b) photograph of the i-TENG. ((c) and (d)) SEM images of micro-patterned PDMS films (c) and nano-structured Al foil (d) in the device. ((e) and (f)) Short-circuit current (e) and open-circuit voltage (f) of the i-TENG when it was implanted under the left chest skin of a rat. Reprinted with permission from Zheng *et al.*, Adv. Mater. **26**(33), 5851 (2014). Copyright 2014 Wiley-VCH.

To increase the electric outputs, certain micro-/nano-patterns can be created on the surface of triboelectric layers. It was demonstrated that the outputs could be raised by 4 to 6 times when micro/nano-patterns were created on the surface of triboelectric layers.^53^ Si wafer with pyramid arrays was often employed as template to fabricate surface features on PDMS. Zheng *et al.* implemented patterned micro-pyramid structures on PDMS surfaces for i-TENG designs (Fig. 7(a)).^50^ A photograph of i-TENG is presented in Fig. 7(b). A PDMS thin film with pyramid micropatterns (Fig. 7(c)) and a nanostructured Al foil (Fig. 7(d)) was utilized as two friction layers. A gold layer-coated Kapton film was used as electrode and supporting substrate. The i-TENG was completely packaged by PDMS to prevent leakage and improve biocompatibility. The i-TENG was implanted in the left chest skin of adult rats. At a constant respiratory rate of 50 times/min, the average I_sc_ and V_oc_ were about 0.14 *μ*A and 3.73 V, which met the voltage range of commercial pacemaker (1.8–2.8 V).

**FIG. 8:**
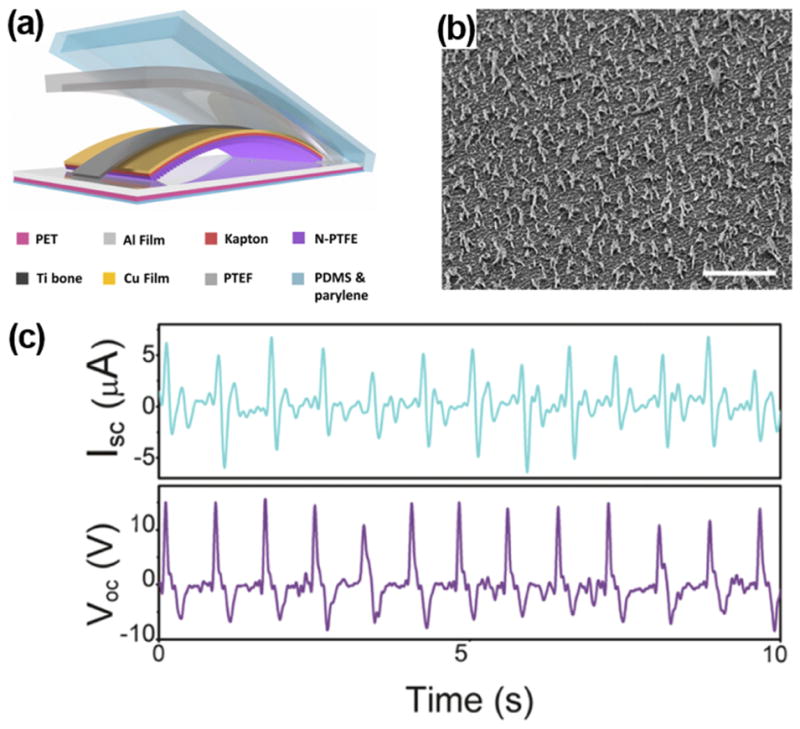
(a) Schematic illustration of i-TENG made from PTFE and Al friction layers. (b) SEM image of nano-structured PTFE films (scale bar: 5 *μ*m). (c) Short-circuit current and open-circuit voltage of the i-TENG implanted between the heart and pericardium of a porcine. Reprinted with permission from Zheng *et al.*, ACS Nano **10**(7), 6510 (2016). Copyright 2016 the American Chemical Society.

Alternatively, Zheng *et al.* used dry etching to create arrays of nanofibers on the PTFE surface for i-TENG development (Fig. 8(a)).^52^ The TENG film was fixed on an arched titanium strip to ensure appropriate elasticity and effective charge transfer (Fig. 8(b)). The entire structure was encapsulated via a core/shell/shell (PTEF/PDMS/Parylene C) configuration, which provided excellent leakage proof. *In vivo* performance of this i-TENG was investigated by fixing it on the heart of a Yorkshire porcine. After the device was set on the inferior wall of the left ventricular, the V_oc_ and I_sc_ of device reached up to 14 V and 5 *μ*A, respectively (Fig. 8(c)). These outputs were the highest among all previously reported i-NGs.

Introducing micro-/nano-patterns to the friction layers allowed higher output under small deformations. This is particularly advantageous for *in vivo* applications, where compact space, low mechanical strains, and limited displacement are commonly encountered. The micro/nano features on the surface of triboelectric layers could notably improve the sensitivity of i-TENG to small mechanical strains. The size of device could be designed smaller to fit in limited spaces inside human body, while remaining at appreciably high outputs. Owing to these unique merits, self-powered IMDs, biosensors, and healthcare/wellness monitors under *in vivo* are mostly designed by i-TENGs with specifically designed surface features.

Although most TENG materials described above are biocompatible, it is still necessary to hermetically package TENGs with nonbiodegradable, biocompatible, and biosafe materials in order to avoid materials erosion, charge leakage, and toxic materials diffusion into the body. In implantable MEMS fabrications, PDMS and Parylene C are widely used packaging or encapsulation materials.^54–56^ PDMS is one of the primary reference materials accepted by the US National Heart, Lung, and Blood Institute, which are discriminatory tools for the evaluation of biomaterials under *in vitro* and *in vivo* conditions. Belanger *et al.* investigated the *in vivo* biocompatibility of PDMS sheets by implanting them intraperitoneally in a rat.^54^ The 12-week results demonstrated that PDMS sheets only induced a very mild inflammatory during the long-term implantation, which was milder than another primary reference material, i.e., low-density polyethylene. Similarly, Parylene C is biologically inert, nonbiodegradable, and mechanically robust, and it has been used as the insulating layer under *in vivo* condition for many years. The i-TENG reported by Zheng *et al.* used Parylene C as the outmost shell in the package.^52^ The biocompatibility of Parylene C was investigated through viability test of mouse fibroblasts (L929 cells). The MTT (3-4,5-dimethylthiazol-2-yl-2,5-diphenyl-2H-tetrazolium bromide) test showed that there was no difference in viability between cells on plates and cells on Parylene C after three days, which demonstrated the excellent biocompatibility of Parylene C.

**FIG. 9:**
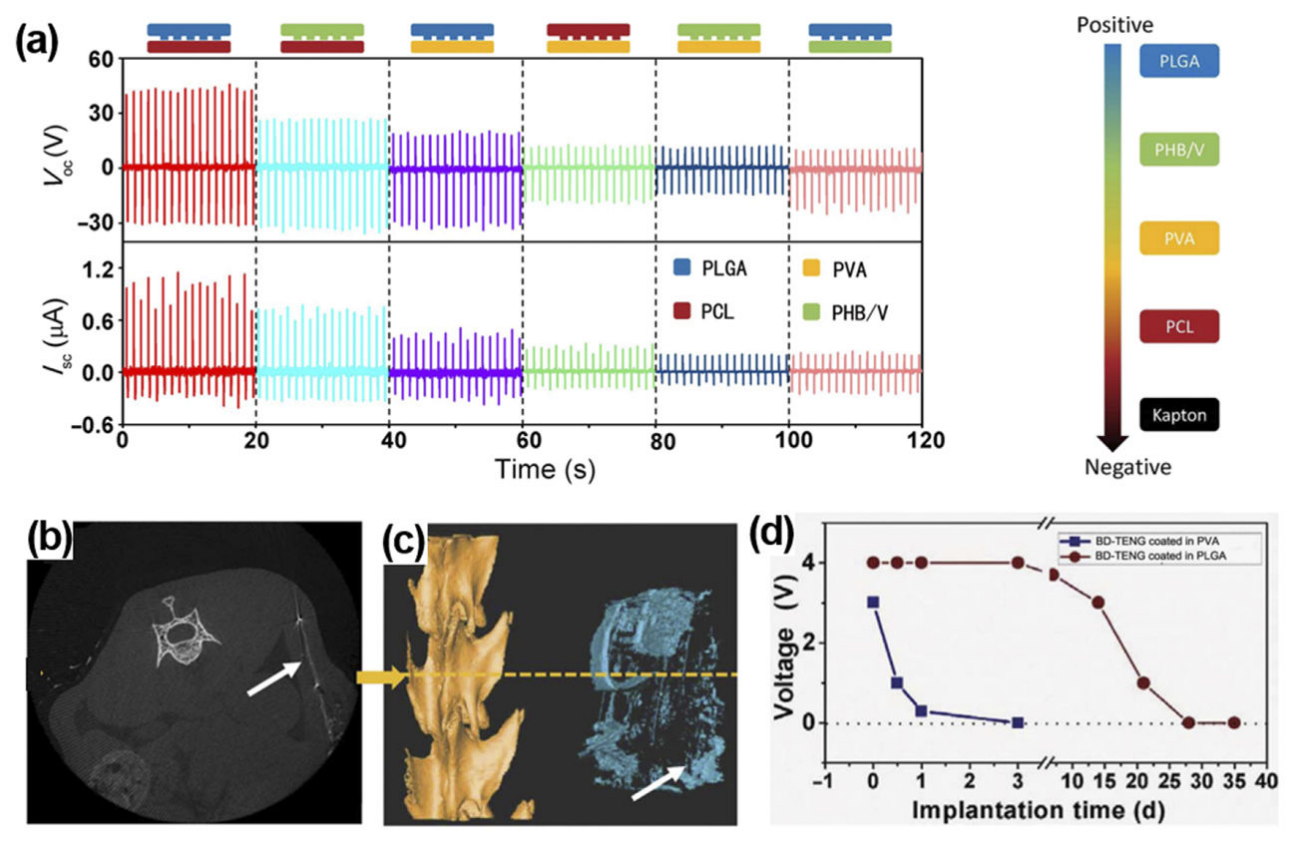
(a) Electrical outputs of TENGs by pairing any two from the four kinds of BDPs as the friction layers. ((b) and (c)) Cross section image (b) and reconstruction image (c) of micro-computer-topography (CT) of BD-TENG after a 9-week implantation. Most parts of the BD-TENGs were biodegraded in the body. (d) Voltage outputs of BD-TENGs coated in PVA and PLGA as a function of time. Reprinted with permission from Zheng *et al.*, Sci. Adv. **2**(3), e1501478 (2016). Copyright 2016 American Association for the Advancement of Science.

Biodegradable polymers (BDPs) could be selected to design i-TENGs for short-term use. Zheng *et al.* fabricated biodegradable TENGs (BD-TENGs) consisting of Mg electrodes and four kinds of BDPs, e.g., poly(L-lactide-co-glycolide) (PLGA), poly(3-hydroxybutyric acid-co-3-hydroxyvaleric acid) (PHB/V), poly(caprolactone) (PCL), and poly(vinyl alcohol) (PVA).^57^ They compared the electric outputs of BD-TENGs made by pairing any two of the four BDPs as the triboelectric layers. The PLGA and PCL pair generated the highest electric outputs with V_oc_ of ~40 V and I_sc_ of ~0.8 *μ*A, owing to their largest difference in electron affinity (Fig. 9(a)). For *in vivo* study, BD-TENGs coated by PLGA and PVA were implanted in subdermal region of a rat. The outputs of PLGA-coated TENG began to decrease after a two-week implantation (Fig. 9(d)). After 9 weeks of implantation, most parts of the BD-TENGs were degraded (Figs. 9(b) and 9(c)) and the wound healed well. The PVA coated TENG exhibited faster degradation. It demonstrated 24-h unaffected operation and was dissolved almost completely within 72 h. The biodegradable i-TENG offers a unique application potential where the electricity is needed temporarily. For example, tissue repairing under the electric stimulation or temporary physiological parameters monitoring post operation. This is a very useful concept for *in vivo* biomechanical energy harvesting with unique application targets. The biodegradable TENGs would be degraded and resorbed by body under designed time periods, reducing the risks of operation and long-term side effects.

In general, material selection and engineering are an essential aspect for i-TENG development. The dielectric material selection and surface engineering determine the electric output, while the packaging materials dictate the life time. For the applications inside biological environment, attentions need to be paid specially to the biocompatibility and biosafety. Long-term *in vivo* examination is desired under TENG operation conditions where appreciable amount of electricity co-exists.

### B. Application and Integration of i-TENGs

The high output, simple configuration, and broad material selection of TENG endowed strong potentials for *in vivo* biomechanical energy harvesting and integration with regular IMDs. So far, a few numbers of i-TENG-powered biomedical systems have been demonstrated, where bulky batteries were replaced by a small and flexible TENG component. In addition, owing to the good sensitivity to physiological actions such as heart beating and respiration, i-TENG could also be designed as self-powered biosensors for healthcare/wellness monitoring.

**FIG. 10:**
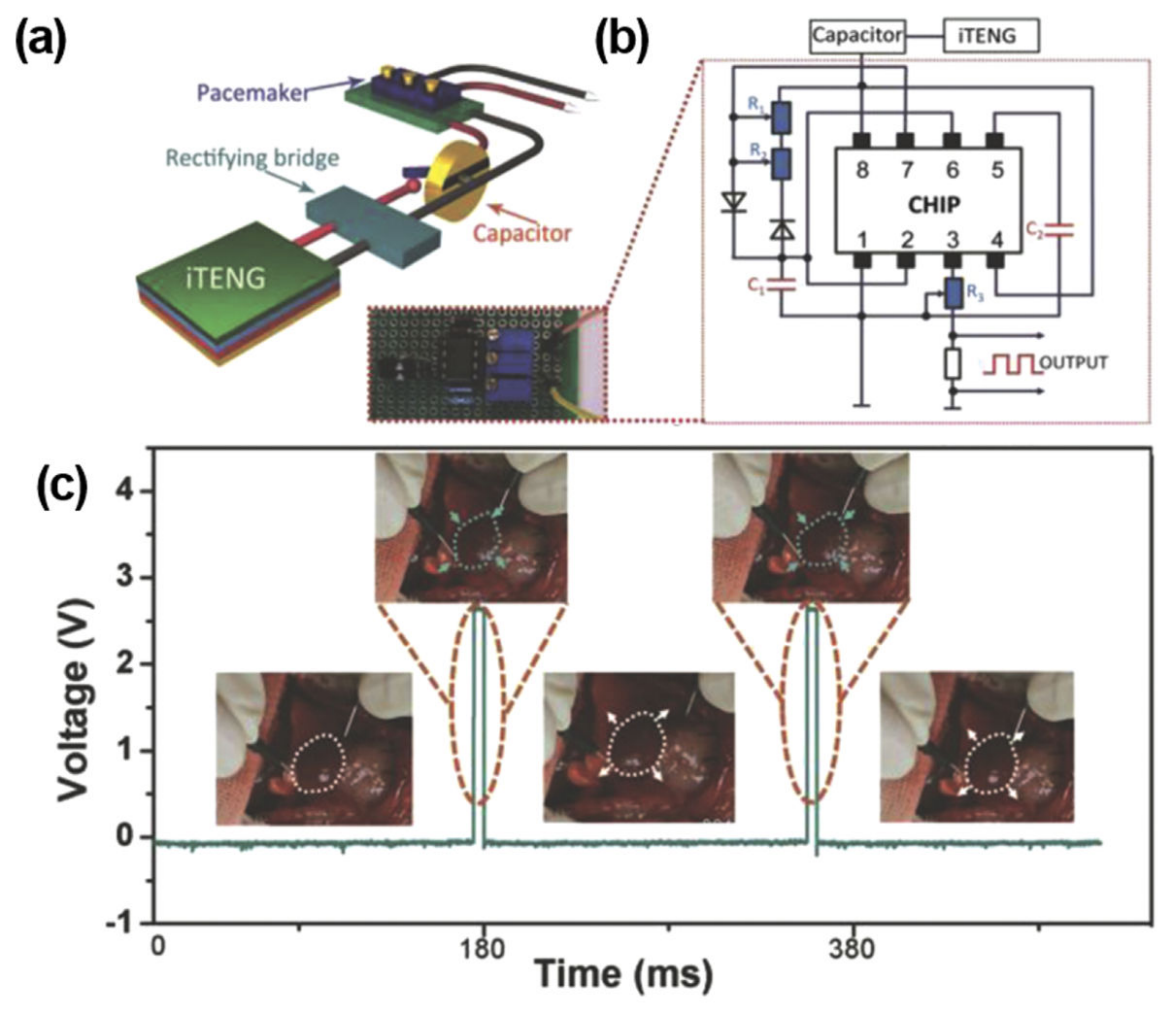
(a) Schematic illustration of a i-TENG-powered pacemaker prototype (b) Electrical circuit of the i-TENG-powered pacemaker prototype. (c) Stimulating voltage generated by the self-powered pacemaker. Insets: regulated heart beating of a rat in response to the pacemaker. Reprinted with permission from Zheng *et al.*, Adv. Mater. **26**(33), 5851 (2014). Copyright 2014 Wiley-VCH.

Pacemaker has been the main target for the self-powering application. Currently, a commercial pacemaker operates at around 3 V,^58^ which is within the range of typical i-TENG outputs. The first i-TENG showed promises to power a pacemaker was demonstrated by Zheng *et al.*^50^ Figure 10(a) shows the schematic diagram of the self-powered pacemaker prototype, composed of an i-TENG, a rectifying bridge, a capacitor, and a pacemaker. The electrical circuit of the prototype was presented in Fig. 10(b). The i-TENG was built from PDMS and Al foil discussed in Sec. III A (Fig. 7(a)). The i-TENG system was implanted under the left chest skin of a rat and converted energy from breathing into ~±3 V AC electricity. The AC outputs were transformed by the full-wave rectifying bridge and stored in a capacitor to drive the pacemaker prototype. The capacitor could be charged from 2 to 3 V within 275 min (equivalent to 13 750 breathing cycles). This amount of energy was sufficient for the pacemaker to generate heart stimulation pulses at various frequencies from 2 to 5 Hz to regulate the heart rates of the rat (Fig. 10(c)).

**FIG. 11:**
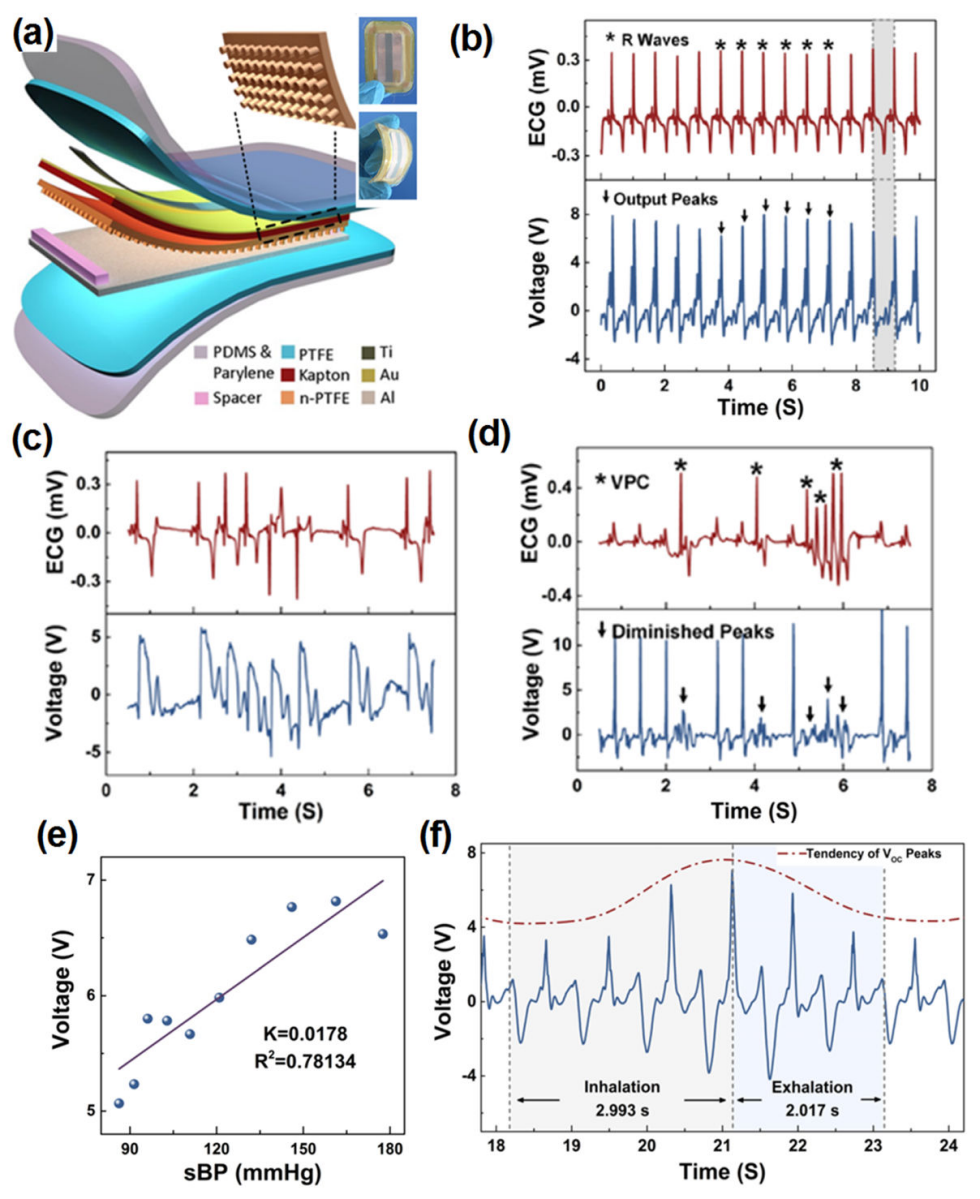
(a) Schematic structure of iTEAS. Inset is a photograph of the iTEAS device. (b) R waves of electrocardiogram and output voltage of the iTEAS. ((c) and (d)) Signals from electrocardiogram and the iTEAS sensor when porcine was in atrial fibrillation (c) and ventricular premature contraction (d). (e) Linear fit of output voltage and blood pressure. (f) Output voltage of iTEAS during a breathing cycle. Reprinted with permission from Ma *et al.*, Nano Lett. **16**(10), 6042 (2016). Copyright 2016 The American Chemical Society.

Essentially, i-TENGs could work individually as self-powered sensors for wellness monitoring *in vivo*. The electric outputs transformed from biomechanics energy contain substantial information related to physiological signals. Ma *et al.* demonstrated a self-powered multifunctional implantable triboelectric active sensor (iTEAS) for healthcare monitoring.^51^ The iTEAS had a similar configuration as thin film-based TENGs as shown in Fig. 11(a). The iTEAS was implanted on the heart of a pig and the electric signal generated was compared to those from electrocardiogram (Fig. 11(b)). An extremely high accuracy of 99% was presented by the sensor, demonstrating the strong potential for heart monitoring function. Based on heart rate monitoring, this device accurately detected irregular rhythms of heartbeats, such as atrial fibrillation (AF) and ventricular premature contraction (VPC), as shown in Figs. 11(c) and 11(d), respectively. The iTEAS was also attempted to monitor blood pressure. There was a linearity (R^2^ = 0.78) with sensitivity of 17.8 mV/mmHg between peak output voltage and blood pressure (Fig. 11(e)). The average velocity of blood flow could be calculated based on a leading time. Besides, respiratory rate was measured by applying the iTEAS to lateral wall of heart. A ratio of the time periods of increasing voltage to those of decreasing voltage was ~1.48 (Fig. 11(f)), which was very close to the inhalation/exhalation ratio. The excellent multi-sensing performance of the iTEAS validated a promising clinical application potential for monitoring and timely diagnosing cardiovascular and chronic respiratory diseases in patients.

## IV. CONCLUSION AND PERSPECTIVES

Since 2006, more than 50 research groups around the world have participated in the study of NGs.^49^ Together with many breakthroughs in NG developments, applications of NG for *in vivo* energy harvesting received tremendous advances during the last decade. As discussed in this review article, both piezoelectric and triboelectric principles have been successfully applied in implantable NG designs. PENG devices are now heavily relying on piezoelectric polymer materials. The device configuration is very simple and has excellent structural integrity. A few volts level output is relatively low but sufficient for low-energy consumption electronics. TENG devices are receiving increasing research interests recently for implantable NG applications. They can produce higher output than PENG as well as possess a broad material selection options. Variable configurations have been proposed and demonstrated for harvesting energy from body motions for powering different functionalities, such as pacemakers and sensors for healthcare monitoring.

Despite the exciting demonstrations, the development of implantable NGs is still in its stage of infancy. For the practical applications, there exist a few critical issues that require to be addressed in the near future:

1. Electric outputs: So far, over 10 V output voltage and micro-ampere output current have been reached by implantable NG prototypes. While this voltage is within the range of operating voltage for most IMDs, the output current or the overall energy still largely falls short to the energy requirement of typical IMDs, such as a pacemaker, which typically operate at a voltage of 3 V and a current of 0.1 mA.^59^ The main reason is that the output from implantable NG is discrete short pulses. New strategies are needed to regulate the electric outputs and raise the overall energy production.
2. Durability of NG devices: So far, the life span investigation of NGs is very briefly conducted. Current reports mostly covered short operation periods in a simulated/artificial environment. It is unknown that how long a NG can survive in a real biological system. For TENG devices that require constant contacts or relative movements, it is extremely important to quantify how long such an effective contact can last, particularly for the devices with micro-/nano-structured surface features. For PENG devices that are made from high-performance ferroelectric materials, it is critical to understand how long the ferroelectric phase would survive in the biological system. Answering these questions requires systematic study in real biological environments over a long period of time.
3. Long-term stability of encapsulation layer: All the NGs for *in vivo* application need to be encapsulated by biocompatible polymers in order to function in the harsh biological environment. Typical encapsulation materials include PDMS and Parylene C. These encapsulation materials can prevent erosion of biofluids and minimize charge leakage. Days and weeks of safe operation have been demonstrated, while practical IMDs require at least years-long life spans. It is necessary to test the stability of encapsulation materials over years. In addition, it should be noted that some biocompatible encapsulation materials such as PDMS would suffer from serious biofouling issues when exposed in a blood contact environment.^60–62^ The hydrophobic surface of PDMS is favorable for the adsorption of proteins and adhesion of cells and bacteria. The fouling effect would significantly degenerate the device performance and thus they are limited in the practical use of IMDs. To overcome the fouling issue, certain surface treatments (e.g., oxygen plasma, ozone, and ultraviolet (UV) light) and modifications (e.g., PEG-based materials and polyzwitterionic antifouling coatings) could be applied to modify the surface hydrophilicity. Nevertheless, most iNGs are packaged by PDMS without any surface modification. The biofouling issue should be seriously investigated in future iNG development for practical IMD applications. Novel package structures (e.g., core/shell and core/shell/shell encapsulation structures) and appropriate modifications of materials may provide better solutions for long-term stable protection for NGs with minimal influence to their energy conversion functionality.
4. Long-term influences to biological systems: Study of how the implantable NGs influence the biological environment, such as contacting tissues, muscles, and organs is just beginning. It is excited to see the negative responses of biological systems to the implanted NGs over a short period of time (a few weeks). This is a good start, while much longer biocompatibility should be further studied in much broader extends. Besides, because operation of NG requires mechanical energy input from the biological system, it is also essential to understand whether the operation of NG would introduce extra burden to the normal body functions. Elastic modulus tuning of NGs marked a good start of this type of research. Nevertheless, much greater research efforts are urgently desired to quantitatively understand the NG’s influences to biological system and minimize the influences through novel materials engineering or system optimization.

In general, using NG to harvest biomechanical energy *in vivo* is an exciting and very promising strategy for powering IMDs. Many research opportunities can be found along this direction. By addressing those critical questions, implantable NGs may eventually find its place as a new power source for future IMDs.

## References

[1] Newman D, Sauve MJ, Herre J, Langberg JJ, Lee MA, Titus C, Franklin J, Scheinman MM, Griffin JC (1992). Am J Cardiol.

[2] Schachter SC, Saper CB (1998). Epilepsia.

[3] Heller A (1999). Annu Rev Biomed Eng.

[4] Meng E, Hoang T (2012). Adv Drug Delivery Rev.

[5] Miller MA, Neuzil P, Dukkipati SR, Reddy VY (2015). J Am Coll Cardiol.

[6] Bazaka K, Jacob M (2012). Electronics.

[7] Wang ZL, Wang XD, Song JH, Liu J, Gao YF (2008). IEEE Pervasive Comput.

[8] Wang ZL (2008). Sci Am.

[9] Qi Y, McAlpine MC (2010). Energy Environ Sci.

[10] Starner T (1996). IBM Syst J.

[11] Shad R, Leland Eli S, Jessy B, Eric C, Elizabeth R, Elaine L, Brian O, Rabaey Jan M, Sundararajan V, Wright Paul K (2005). IEEE Pervasive Comput.

[12] Wang ZL, Song J (2006). Science.

[13] Wang X, Liu J, Song J, Wang ZL (2007). Nano Lett.

[14] Niu S, Wang X, Yi F, Zhou YS, Wang ZL (2015). Nat Commun.

[15] Tang W, Tian J, Zheng Q, Yan L, Wang J, Li Z, Wang ZL (2015). ACS Nano.

[16] Wang J, Li S, Yi F, Zi Y, Lin J, Wang X, Xu Y, Wang ZL (2016). Nat Commun.

[17] Yang PK, Lin L, Yi F, Li X, Pradel KC, Zi Y, Wu CI, He JH, Zhang Y, Wang ZL (2015). Adv Mater.

[18] Hansen BJ, Liu Y, Yang R, Wang ZL (2010). ACS Nano.

[19] Yang R, Qin Y, Li C, Zhu G, Wang ZL (2009). Nano Lett.

[20] Zhu R, Zhang WG, Yang RS (2012). Sci Adv Mater.

[21] Wang XD (2012). Nano Energy.

[22] Wang ZL (2013). ACS Nano.

[23] Briscoe J, Dunn S (2015). Nano Energy.

[24] Hu F, Cai Q, Liao F, Shao M, Lee ST (2015). Small.

[25] Zhang H, Zhang XS, Cheng XL, Liu Y, Han MD, Xue X, Wang SF, Yang F, Smitha AS, Zhang HX, Xu ZY (2015). Nano Energy.

[26] Li Z, Zhu G, Yang R, Wang AC, Wang ZL (2010). Adv Mater.

[27] Yuan M, Cheng L, Xu Q, Wu W, Bai S, Gu L, Wang Z, Lu J, Li H, Qin Y, Jing T, Wang ZL (2014). Adv Mater.

[28] Dagdeviren C, Yang BD, Su Y, Tran PL, Joe P, Anderson E, Xia J, Doraiswamy V, Dehdashti B, Feng X, Lu B, Poston R, Khalpey Z, Ghaffari R, Huang Y, Slepian MJ, Rogers JA (2014). Proc Natl Acad Sci U S A.

[29] Lu B, Chen Y, Ou D, Chen H, Diao L, Zhang W, Zheng J, Ma W, Sun L, Feng X (2015). Sci Rep.

[30] Cheng L, Yuan MM, Gu L, Wang Z, Qin Y, Jing T, Wang ZL (2015). Nano Energy.

[31] Cheng XL, Xue X, Ma Y, Han MD, Zhang W, Xu ZY, Zhang H, Zhang HX (2016). Nano Energy.

[32] Yu YH, Sun HY, Orbay H, Chen F, England CG, Cai WB, Wang XD (2016). Nano Energy.

[33] Yang R, Qin Y, Dai L, Wang ZL (2009). Nat Nanotechnol.

[34] Zhou J, Xu NS, Wang ZL (2006). Adv Mater.

[35] Li Z, Yang RS, Yu M, Bai F, Li C, Wang ZL (2008). J Phys Chem C.

[36] Bowen CR, Kim HA, Weaver PM, Dunn S (2014). Energy Environ Sci.

[37] Qi Y, Jafferis NT, Lyons K, Lee CM, Ahmad H, McAlpine MC (2010). Nano Lett.

[38] Chen X, Xu S, Yao N, Shi Y (2010). Nano Lett.

[39] Wu W, Cheng L, Bai S, Dou W, Xu Q, Wei Z, Qin Y (2013). J Mater Chem A.

[40] Inaoka T, Shintaku H, Nakagawa T, Kawano S, Ogita H, Sakamoto T, Hamanishi S, Wada H, Ito J (2011). Proc Natl Acad Sci U S A.

[41] Cha S, Kim SM, Kim H, Ku J, Sohn JI, Park YJ, Song BG, Jung MH, Lee EK, Choi BL, Park JJ, Wang ZL, Kim JM, Kim K (2011). Nano Lett.

[42] Wang YR, Zheng JM, Ren GY, Zhang PH, Xu C (2011). Smart Mater Struct.

[43] Mao YC, Zhao P, McConohy G, Yang H, Tong YX, Wang XD (2014). Adv Energy Mater.

[44] Sun CL, Shi J, Bayerl DJ, Wang XD (2011). Energy Environ Sci.

[45] Chang C, Tran VH, Wang J, Fuh YK, Lin L (2010). Nano Lett.

[46] Laroche G, Marois Y, Guidoin R, King MW, Martin L, How T, Douville Y (1995). J Biomed Mater Res.

[47] Zhang ZY, Yao CH, Yu YH, Hong ZL, Zhi MJ, Wang XD (2016). Adv Funct Mater.

[48] Fan FR, Tian ZQ, Wang ZL (2012). Nano Energy.

[49] Wang ZL, Chen J, Lin L (2015). Energy Environ Sci.

[50] Zheng Q, Shi B, Fan F, Wang X, Yan L, Yuan W, Wang S, Liu H, Li Z, Wang ZL (2014). Adv Mater.

[51] Ma Y, Zheng Q, Liu Y, Shi B, Xue X, Ji W, Liu Z, Jin Y, Zou Y, An Z, Zhang W, Wang X, Jiang W, Xu Z, Wang ZL, Li Z, Zhang H (2016). Nano Lett.

[52] Zheng Q, Zhang H, Shi B, Xue X, Liu Z, Jin Y, Ma Y, Zou Y, Wang X, An Z, Tang W, Zhang W, Yang F, Liu Y, Lang X, Xu Z, Li Z, Wang ZL (2016). ACS Nano.

[53] Fan FR, Lin L, Zhu G, Wu W, Zhang R, Wang ZL (2012). Nano Lett.

[54] Belanger MC, Marois Y (2001). J Biomed Mater Res.

[55] Chang TY, Yadav VG, De Leo S, Mohedas A, Rajalingam B, Chen CL, Selvarasah S, Dokmeci MR, Khademhosseini A (2007). Langmuir.

[56] Irimia-Vladu M (2014). Chem Soc Rev.

[57] Zheng Q, Zou Y, Zhang Y, Liu Z, Shi B, Wang X, Jin Y, Ouyang H, Li Z, Wang ZL (2016). Sci Adv.

[58] Hwang GT, Park H, Lee JH, Oh S, Park KI, Byun M, Park H, Ahn G, Jeong CK, No K, Kwon H, Lee SG, Joung B, Lee KJ (2014). Adv Mater.

[59] Southcott M, MacVittie K, Halamek J, Halamkova L, Jemison WD, Lobel R, Katz E (2013). Phys Chem Chem Phys.

[60] Zhang H, Chiao M (2015). J Med Biol Eng.

[61] Wong I, Ho CM (2009). Microfluid Nanofluid.

[62] Makamba H, Kim JH, Lim K, Park N, Hahn JH (2003). Electrophoresis.

